# Climate Change, Heat Stress, and Kidney Disease–Associated Mortality and Health Care Utilization

**DOI:** 10.1016/j.ekir.2024.08.018

**Published:** 2024-08-22

**Authors:** Naresh Kumar, Venkata Madhavi Latha Telagarapu, Alessia Fornoni

**Affiliations:** 1 Miller School of Medicine, University of Miami, Miami Florida, USA; 2 Peggy and Harold Katz Family Drug Discovery Center, Miller School of Medicine, University of Miami, Miami Florida, USA

See Clinical Research on Page 2946

Climate change is exacerbating our exposure to chronic heat stress due to a gradual increase in ambient temperature and relative humidity, and acute heat stress due to intensifying frequency and duration of heat waves. Both chronic and acute heat stress exposures pose unprecedented threat to public health. When the body’s core temperature exceeds 37 ± 0.5 °C, hypothalamus initiates thermoregulatory processes to dissipate heat through radiation and evaporative cooling. Heat radiation is inefficient when air temperature is higher than the body’s core temperature. Therefore, thermal homeostasis is achieved through evaporative cooling by peripheral vasodilation to increase blood flow to the skin, sweat secretion, and cardiac output by hypotension, which limits blood flow to the vital organs by arteriole hypertension and impairs their functions, including renal function (Figure 1).^1^ Therefore, patients with chronic kidney disease (CKD) are particularly at risk of multiple adverse events, including acute kidney injury (AKI) and stroke. Heat stress is increasingly recognized as a risk factor of CKD of unknown origin affecting outdoor workers.^2^ Using the nationally representative data, Xi *et al.*^3^ documented elevated risk of mortality and health care utilization among patients on hemodialysis under moderately high temperature (≥ 29.5 °C) in the US. This research contributes to the growing body of literature on the increased health risks of heat stress in vulnerable population. However, etiology-specific analysis can enhance the clinical implications of the study.

**Figure 1:**
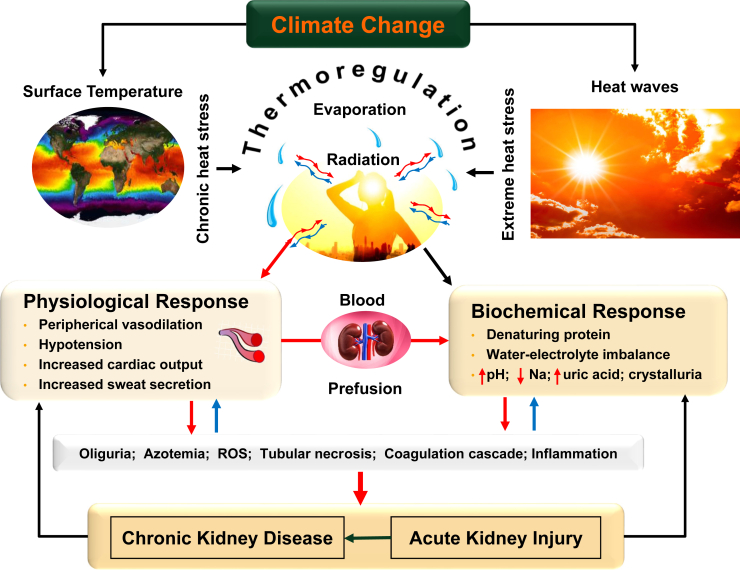
A conceptual framework of the relationship between climate, heat stress, and kidney diseases. Na, sodium; ROS, reactive oxygen species.

### Acute Heat Stress–Related Health Risks

The highest risk of emergency room visit was observed for all-cause and cardiovascular events in the patients on hemodialysis when the average ambient temperature was ≥ 99th percentile, that is, ≥ 32.3 °C.^3^ Ambient temperature alone does not account for relative humidity, which greatly affects evaporative cooling; for example, a 33.3 °C ambient temperature with a 65% relative humidity feels like (or a heat index of) 42.2 °C.^4^ Therefore, heat index is clinically more relevant than the dry bulb ambient temperature. When heat index is used, the relative-risk of all-cause mortality in similar patients on hemodialysis was 18% higher when 2-day or 1-day lagged heat exposures were ≥ 40.6 °C or ≥ 46.1 °C,^5^ respectively, as compared to 7% higher mortality risk reported by Xi *et al.*^3^

Prolonged exposure to acute heat stress compromises the body’s thermoregulatory ability (Figure 2a), which can result in multiple organ failures, including stroke and AKI, and frequent AKI can also result in onset of CKD.^6^ Because heat stress not only denatures and inactivates cellular proteins^7^ and upregulates coagulation pathways,^8^ it also severely distorts water and electrolyte imbalance followed by a cascade of pathological changes (Figure 1). Vulnerable populations, including young children, elderly, obese individuals, and people with preexisting health conditions, must be protected from prolonged exposure to heat stress because of their impaired thermoregulation ability to dissipate heat. Outdoor workers, especially in tropical climate, are at the greatest health risk of acute heat stress exposure due to their routine direct sun exposure for many hours, which is likely to be an important risk factor of CKD of unknown origin.^2^ Not only do they need to dissipate incoming radiation, but they also need to dissipate heat generated by their working muscles. This extreme heat stress makes them susceptible to the risk of both AKI and CKD. Thus, it is important to tease out the relative risk of mortality and health care utilization by age and occupation in response to heat stress.

**Figure 2:**
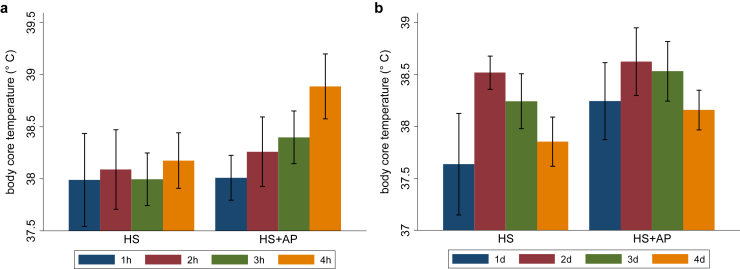
Animals’ ability to regulate their body’s core temperature in response to the duration and frequency of heat stress exposure. In a murine experiment, 8- to 9-week-old male rats (*n* = 5) and rats previously exposed to real-world particulate air pollution (*n* = 6) were exposed to heat stress for 4 hours with a 15-minute rest break consecutively for 4 days. Animals were allowed to recover for 12 hours after the exposure on each day and their core body temperature was measured; (a) core body temperature by duration of exposure in hours (1–2-hour temperature ∼38.01 °C vs. 3–4-hour temperature ∼38.8 °C; difference 0.29 °C, *P* < 0.01); (b) core body temperature by frequency of exposure, i.e., number of days (2nd day temperature ∼38.3 °C; 4th day temperature ∼37.3 °C; difference ∼1.02 °C; *P* < 0.001). AP, air pollution; HS, heat stress.

### CKD and Increasing Surface Temperature

A gradual increase in global surface temperature due to climate change, for example, 1 °C to 2 °C is likely to exacerbate the risk of CKD. To evaluate the implications of such changes, observational studies need to evaluate the risk of CKD with respect to continuous long-term distributed lagged heat exposure instead of extreme hyperthermic conditions. Modelling the relative risk of mortality or hospital visit or incidence of new CKD or changes in kidney function with respect to a unit increase in the distributed time-lagged exposure to heat stress for 1 to 3 years at intervals of a week or month will shed light on the increasing burden of CKD due to chronic heat stress. The analysis also needs to factor in the number of extreme weather events during the exposure periods; these events exacerbate multiple environmental stressors and interrupt health care delivery,^9^ which can cause AKI and its subsequent progression to CKD.

### Region- and Season-Specific Health Effects of Heat Stress

Health risk of heat stress varies by climate-region due to long-term region-specific and short-term season acclimation or adaptations.^10^^,^^11^ Xi *et al.*^3^ contribute to the growing literature on region-specific variations in the health effects of heat stress.^5^^,^^11^ The role of acclimation in the health risks of heat stress warrants further research. Animals’ ability to regulate their body’s core temperature improved as they acclimatized to heat stress (Figure 2b). Acclimation under moderate hyperthermic conditions upregulated heat shock protein 70, which reduced the risk of kidney injury when exposed to hyperthermic conditions (temperature ≥ 40 °C and 40% relative humidity).^12^ Epidemiological studies further substantiate these findings suggesting that people living in a hot and humid climate in the US had lower risk of different diseases, such as multiple sclerosis and allergic conjunctivitis than those living in colder climate.^10^^,^^11^ However, prior exposure to other adverse environmental conditions, such as air pollution, can exacerbate the effects of heat stress (Figure 2a and b). These findings warrant clinical interventions to explore the role of heat and heat-cold therapies in enhancing people’s ability to better adapt to heat stress.

### Clinical and Public Health Response

Global warming is exacerbating exposure to multiple climate-sensitive environmental stressors (CSES), such as heat stress, aeroallergens, and air pollutants. This warrants an urgent clinical and public health response to prepare and adapt for the anticipated changes in CSES. First, an integrated real-time surveillance is warranted. Such surveillance can provide all stakeholders, including clinicians, patients, the public, and decision makers, with information regarding the levels and types of CSES (e.g., the weather) at a given location and time and their associated risk of a disease; these should be easily accessible through multimedia platforms. Of course, this will require transdisciplinary collaboration to acquire real-time weather, health, and socio-demographic data, classical epidemiological research, emerging time-space dynamic machine learning and artificial intelligence algorithms for health risk prediction, along with testing and validation of their prediction efficacy. Some patients already manage their health risk using electronic devices, such as Apple watches or Fitbits; however, most of these devices are restricted to physiological measurement and/or biometric measurements and lack access to CSES data. Second, health care professionals need to be trained in translating CSES health risk information into their clinical practice and health care delivery. For example, they need to use the anticipated heat stress, air pollution, and aeroallergen information to prepare their patients to avoid, mitigate, and counteract the adverse health risks of CSES in the coming days, weeks, and months. Some of these measures could be as simple as hydration, avoiding work or sports during the peak hours of the day, and proactive access to preventive medical therapies. Finally, it is necessary to prepare for prompt and proactive health care delivery in response to the anticipated short-term and long-term changes in CSES, whether it is a heat wave event or the aftermath of a hurricane. For example, after hurricanes, people suffer from multiple CSES, including contaminated water, heat stress, and severe interruption in health care delivery.^9^ Patients on hemodialysis are particularly vulnerable in the aftermath of a hurricane; however, mobile health care delivery can address this unmet need of such patients.

## Disclosure

All the authors declared no competing interests.
